# Supplementary material for: Warming proportional to cumulative carbon emissions not explained by heat and carbon sharing mixing processes
Source: Nat Commun. 2023 Oct 13;14:6466. doi: 10.1038/s41467-023-42111-x (PMC10576026; doi:10.1038/s41467-023-42111-x)
Supplement: Supplementary file 1 — Supplementary Information [file 41467_2023_42111_MOESM1_ESM.pdf]

Supplementary Information for

Warming proportional to cumulative carbon emissions not explained by heat and carbon sharing mixing processes

Nathan P. Gillett[1],

[1]Canadian Centre for Climate Modelling and Analysis, Environment and Climate Change Canada, Victoria, BC, Canada.

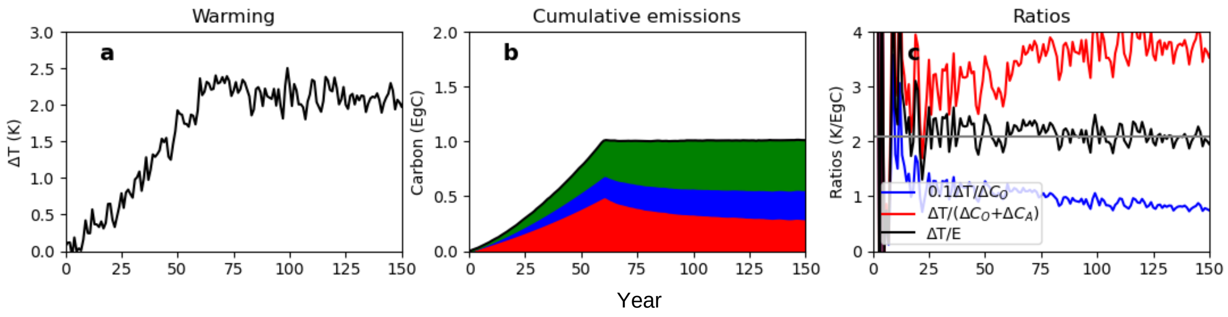

Supplementary Fig. 1 Comparison of warming, cumulative carbon emissions and their ratio in a zero emissions simulation from CanESM5. As Figure 2, but showing warming, cumulative emissions and their ratio in a simulation in which atmospheric CO_2 concentration increases at 1% per year (1pctCO2)[14] up until the time at which cumulative emissions reach 1000 PgC (year 61), after which emissions cease, and atmospheric CO_2 concentration is allowed to evolve freely. This experiment is the esm-1pct-brch-1000PgC experiment of the Zero Emissions Commitment Model Intercomparison Project[21] (ZECMIP). While this experiment was in fact initialized at year 61 of a simulation in which large-scale CO_2 was relaxed towards the 1pctCO2 mean concentration, the output from this relaxed-CO_2 experiment was never published, therefore we instead use output from the prescribed-CO_2 1pctCO2 and piControl simulations for this plot, but this should result in only negligible differences in the results shown here. Panel **a** shows global mean near-surface air temperature anomalies relative to the preindustrial control in black. Panel **b** shows corresponding changes in atmosphere (red), ocean (blue) and land (green) carbon pools, in EgC, and their sum (black line), which is equal to diagnosed cumulative CO_2 emissions. Panel **c** shows the ratio in K/EgC of the warming to the increase in ocean carbon (scaled by a factor of 0.1 for display purposes; blue), the ratio of the warming to the increase in ocean plus atmosphere carbon (red), and the ratio of the warming to cumulative CO_2 emissions (i.e. the increase in ocean plus atmosphere plus land carbon; black). The grey line shows CanESM5's Transient Climate Response to Emissions (TCRE).

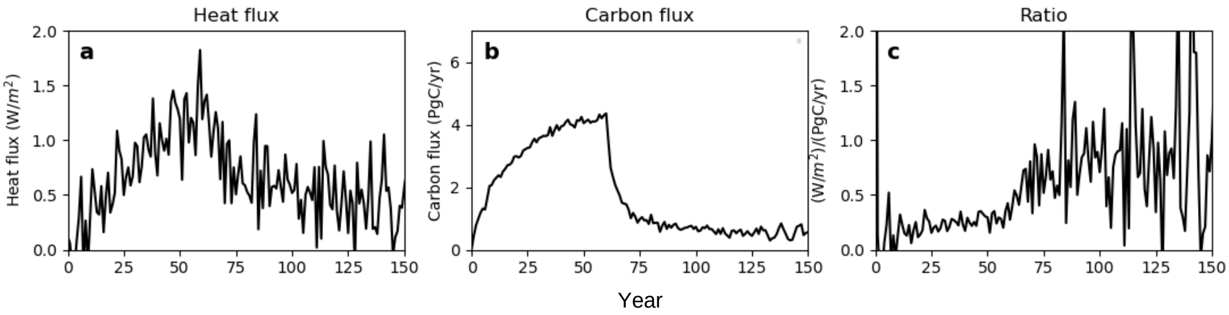

Supplementary Fig. 2 Atmosphere-ocean heat flux, atmosphere-ocean carbon flux and the ratio of heat to carbon flux in in a zero emissions simulation from CanESM5. As Figure 3, but showing global mean atmosphere-ocean heat flux anomalies relative to preindustrial control (**a**), global total atmosphere-ocean carbon flux anomalies relative to preindustrial control (**b**), and the ratio of heat flux to carbon flux anomalies (**c**) in the 1pctCO2 and esm-1pct-brch-1000PgC simulations from CanESM5, which are described in the caption to Supplementary Fig. 1.
